# Small bowel obstruction secondary to primary enterolith: A rare and delayed complication of radiation enteritis

**DOI:** 10.1002/ccr3.3700

**Published:** 2021-01-16

**Authors:** Charalampos Seretis, Lucy Archer, Mohammed Ali Elhassan, Deborah Gurung, Amitabh Palit, Kassim Zayyan

**Affiliations:** ^1^ Department of General Surgery George Eliot Hospital NHS Trust Nuneaton UK; ^2^ Department of Radiology George Eliot Hospital NHS Trust Nuneaton UK

**Keywords:** emergency, enterolith, obstruction, radiation, surgery

## Abstract

Primary enteroliths as a result of pelvic radiotherapy are a rare cause of intestinal obstruction.

## Graphical abstract

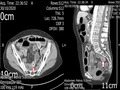

## 1 CASE DESCRIPTION

Primary enteroliths are an extremely rare cause of intestinal obstruction and can represent a late complication of radiation enteritis, with the mechanism of formation being chronic enteric content stasis due to reactive small bowel thickening induced by the radiotherapy. High clinical suspicion and radiological confirmation aid establishing the diagnosis.

**FIGURE 1:**
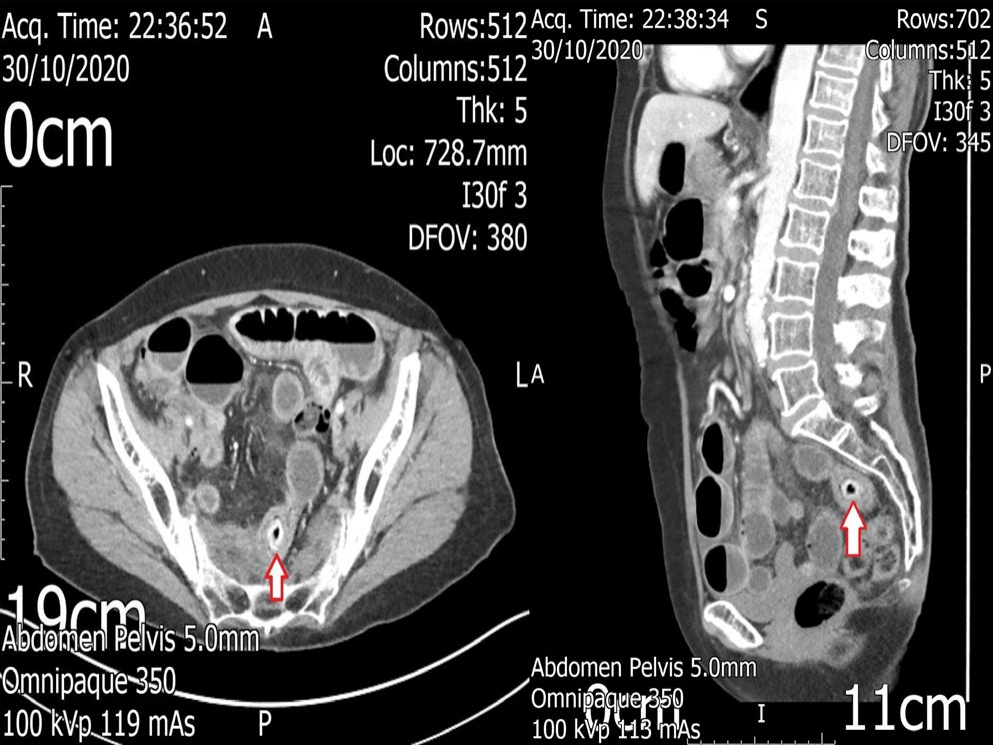
Axial and sagittal CT images of the patient, with evident small bowel obstruction secondary to the impacted enterolith within a thickened ileal loop (red arrows)

A 73‐year old Caucasian female patient presented to our acute surgical take with features of gastrointestinal obstruction, comprising of abdominal distension, vomiting, and sharp pain localized in the pelvic region. Her past medical history comprised of a course of radiotherapy for early cervical cancer, approximately 10 years prior, with the patient being cleared from subsequent oncology follow‐up due to complete response. The patient mentioned complete inability to tolerate oral intake and described previous similar episodes of intermittent intestinal obstruction over the previous year. Clinical examination revealed a moderately distended but soft abdomen, with tenderness in deep palpation in the whole of lower abdomen; no obvious external hernia was identified. Provisional diagnosis was that of intestinal obstruction either due to congenital adhesions or radiation enteritis causing intestinal stricture and hence an urgent abdominopelvic computed tomography (CT) scan with intravenous contrast was performed for further assessment. The performed CT scan revealed the presence of dilated small bowel loops and identified a transition point in the lower abdomen, with a concurrent intraluminal filling defect at the site of small bowel caliber change (Figure 1). The patient was scheduled for emergency laparotomy, and intraoperatively, a solid enterolith was found impacted within a thickened but patent ileal segment as cause of the intestinal obstruction. The impacted 2.5 cm enterolith was milked more proximally and was extracted through a 3 cm enterotomy, which was subsequently closed in a double‐layer fashion. Unfortunately, an intraoperative picture was not taken as the patient was not consented for that prior to the procedure. The patient had an uneventful recovery and was discharged in a stable condition, after resumption of gastrointestinal function.

Our case is one of the very few in the literature describing acute gastrointestinal obstruction from a primary enterolith as a delayed complication of small bowel radiation‐induced enteritis.^1^, ^2^ Detailed history with high index of clinical suspicion in slimier cases, along with and prompt investigation with cross‐sectional abdominopelvic imaging, is mandated to establish this extremely rare diagnosis and facilitate further surgical planning.

## CONFLICT OF INTEREST

The authors declared no potential conflicts of interest with respect to the research, authorship, and/or publication of this article.

## AUTHOR CONTRIBUTIONS

CS and LA: contributed to the clinical data collection and prepared the case report; MAE and DG: contributed to the design of the case report presentation; AP and KZ: performed the final revision of the manuscript.

## ETHICAL APPROVAL

Informed consent was obtained from the patient and is available upon request by the editorial office; no ethical committee approval was required for the publication of this case report.

## Data Availability

The presented data are part of the manuscript.

## References

[1] Singh MP , Huda T , Singh KV . A primary jejunal enterolith presenting as small bowel obstruction. Indian J Surg. 2018;80(3):292‐293. 10.1007/s12262-018-1753-0 29973766 PMC6014940

[2] Gurvits GE , Lan G . Enterolithiasis. World J Gastroenterol. 2014;20(47):17819‐17829. 10.3748/wjg.v20.i47.17819 25548480 PMC4273132

